# Supplementary material for: Acute activation of Gq-signaling in islet macrophages inhibits β-cell insulin secretion through AMPK-sphingolipid axis
Source: bioRxiv. 2025 Oct 30:2025.10.29.680858. Preprint. [Version 1] doi: 10.1101/2025.10.29.680858 (PMC12636625; doi:10.1101/2025.10.29.680858)
Supplement: Supplement 1 [file NIHPP2025.10.29.680858v1-supplement-1.pdf]

bioRxiv preprint doi: https://doi.org/10.1101/2025.10.29.680858; this version posted October 30, 2025. The copyright holder has placed this preprint (which was not certified by peer review) in the Public Domain. It is no longer restricted by copyright. Anyone can legally share, reuse, remix, or adapt this material for any purpose without crediting the original authors.

Supplementary Figures:

Supp Figure 1. Gq/11 expression in mouse macrophages and human PBMCs. (A) Integrated UMAP of scRNA seq from murine islets from published datasets from RC, HFD and Obese (B) Violin plots of marker gene expression from scRNA seq datasets of islet cell types in mice fed regular chow (RC), high-fat diet (HFD), or db/db background. (C) Major immune cell subsets (macrophages, T cells, B cells) across RC, HFD, and db/db mice, represented as percentage of population. (D) *Gna11* expression in bone marrow–derived macrophages (BMDMs) from RC and HFD mice (n=5-8). (E) Correlation of *Gna11* expression in peritoneal macrophages (PMs) with body weight (n=10). (F) Expression of *Gnaq* and *Gna11* in PMs from RC versus HFD mice (n=5). (G-H) Correlation of *Gnaq* expression in PMs with (G) body weight and (H) blood glucose (n=9-10). (I-J) Correlation of *Gna11* expression in PMs with (I) body weight and (J) blood glucose (n=9-10). (L-N) Human PBMC analysis: *GNAQ* expression correlated with (L) blood glucose, and *GNA11* expression with (M) body mass index (BMI) and (N) fasting glucose (n=12-18). Data represented as mean ± s.e.m.; Pearson's correlation shown for E, G–J, K–N. *$p < 0.01$, $p < 0.05$, NS = not significant.

Supp Figure 2. Glucose homeostasis and hormone regulation in LysM-GqD and control mice under regular chow (RC) and high-fat diet (HFD) condition. (A) Body weight of LysM-GqD and control mice maintained on RC (n=7-11). (B) Intraperitoneal glucose tolerance test (GTT) (n=10) and (C) Glucose-stimulated insulin secretion (GSIS) in RC-fed LysM-GqD and control mice without DCZ stimulation (n=6). (D) GTT in RC-fed LysM-GqD and control mice after acute DCZ (10 µg/kg) stimulation (n=10). (E) Pyruvate tolerance test (PTT) in RC-fed LysM-GqD and control mice with DCZ (30 µg/kg) stimulation (n=7-11). (F) Body weight progression of LysM-GqD and control mice under HFD (n=9-11). (G) PTT in HFD-fed LysM-GqD and control mice with DCZ (30 µg/kg) stimulation (n=9). (H) Oral glucose tolerance test (O-GTT) in HFD-fed LysM-GqD and control mice with DCZ (30 µg/kg) stimulation (n=6-9). Data represented as mean ± s.e.m. using One-tailed unpaired t-test (A-H); *$p < 0.05$, **$p < 0.01$, ***$p < 0.001$; NS = not significant.

Supp Figure 3. Clodronate liposome treatment effectively depletes islet macrophages. (A) Quantification of F4/80$^+$ mRNA expression in islets after clodronate liposome treatment compared with control liposomes (DAPI, blue; F4/80, green), scale-20µM (n = 3). (B) Immunoblot for HA-tag confirming expression of LysM-GqD in macrophages; β-actin serves as loading control. (C) Representative immunofluorescence images of pancreatic islets stained with DAPI (blue) and F4/80 (green) in control versus clodronate liposomes–treated mice. White arrowheads indicate

bioRxiv preprint doi: https://doi.org/10.1101/2025.10.29.680858; this version posted October 30, 2025. The copyright holder has placed this preprint (which was not certified by peer review) in the Public Domain. It is no longer restricted by copyright. Anyone can legally share, reuse, remix, or adapt this material for any purpose without crediting the original authors.

F4/80^{+} macrophages, which are markedly reduced after clodronate treatment (n=3). Data represented as mean ± s.e.m.; ***p < 0.001.

Supp Figure 4. Myeloid Gq Activation Does Not Alter Whole-Body Energy Expenditure or Substrate Utilization both in RC and HFD condition. (A–D) Energy expenditure in RC-fed mice: (A) Time-course of total energy expenditure across light and dark cycles (n=5-6); (B–D) Quantification of resting, active, and total energy expenditure showing no significant differences between genotypes (n=5-6). (E–H) RER in RC-fed mice: (E) RER traces across light and dark cycles; (F–H) Quantification of resting, active, and total RER, indicating comparable substrate utilization between groups. (I–L) Energy expenditure in HFD-fed mice: (I) Time-course of total energy expenditure; (J–L) Quantification of resting, active, and total energy expenditure showing no genotype-dependent differences. (M–P) RER in HFD-fed mice (n=6): (M) RER time-course across light and dark cycles; (N–P) Quantification of resting, active, and total RER, with no significant differences between groups. Data are presented as mean ± s.e.m. using One-tailed unpaired t-test; NS = not significant.

Supp Figure 5. Lipidomic profiling reveals dynamic lipid remodeling in LysM-GqD macrophages. (A–B) Principal component analysis (PCA) (A) and volcano plots (B) of cell pellet lipids at 0, 1, and 2 hours after DCZ stimulation, showing distinct clustering and significant lipid changes at 2 hours. (C) Pie chart illustrating major lipid pathways significantly altered after 2 hours of DCZ treatment. (D) Distribution of lipid classes contributing to differential regulation. (E) Heatmap depicting differentially regulated lipid species at 2 hours in control versus LysM-GqD macrophages. (F) Pathway enrichment analysis identifying lipid metabolic pathways significantly modulated upon Gq activation. (G) PLSDA and (H) volcano plots of lipid changes in cell supernatants comparing control vs LysM-GqD cells following DCZ stimulation. (I) PLSDA and (J) Volcano plot comparing lipid changes between control and LysM-GqD macrophages after Cpd C treatment. All the experiments had 3 biological independent biological replicates and 2 technical replicates for each sample.

Supp Figure 6. Glucose and lipid metabolic profiling in LysM-Gq KO mice. (A–D) Glucose tolerance test (GTT) (n=6-7) (A), insulin tolerance test (ITT) (n=6-7) (B), pyruvate tolerance test (PTT) (n=6-7) (C), and oral glucose tolerance test (O-GTT) (n=6-7) (D) in control and LysM-Gq KO mice. Knockout mice show modest differences in glucose handling, with largely comparable overall glucose excursions (AUC). (E–G) Plasma profiling in fed and fasted states. Plasma glycerol levels were significantly altered in KO mice (n=7-8) (E), whereas plasma triglycerides

bioRxiv preprint doi: https://doi.org/10.1101/2025.10.29.680858; this version posted October 30, 2025. The copyright holder has placed this preprint (which was not certified by peer review) in the Public Domain. It is no longer restricted by copyright. Anyone can legally share, reuse, remix, or adapt this material for any purpose without crediting the original authors.

(n=7-9) (F) and free fatty acids (n=7-9) (G) remained unchanged. (H) *Gnaq* expression levels in BMDMs from control and LysM-Gq KO mice (n=3). Data represented as mean ± s.e.m. using One-tailed unpaired t-test (A-G); *p < 0.05, **p < 0.01, ***p < 0.001; NS = not significant.

Supp Figure 7. Gpr18 expression and functional assessment in islet macrophages and LysM-Gq KO models. (A) Relative GPCRs expression profiling in murine islet macrophages reveals Gpr18 enrichment. (B) Analysis of publicly available single-cell RNA seq datasets confirms immune cell specific expression of Gpr18 in murine islets. (C–E) Gpr18 expression in macrophages is reduced under HFD conditions (C) and inversely correlates with body weight (D) and blood glucose levels (E). (F) IP-One assay demonstrating NAGly (GPR18 agonist)–induced, dose-dependent IP1 accumulation in DCZ-stimulated GqD macrophages. (G–I) Body weight analysis showing no significant differences between control and NAGly-treated mice (n=8) (G), LysM-Gq KO and control mice (n=8) (H), and LysM-PTX and control mice (n=8) (I). (J–L) Coculture of BMDM and INS-1 cell for insulin secretion assays in control and LysM-Gq KO islets following stimulation with glyburide (n=4) (J), arginine (n=4) (K), or Exendin-4 (n=4) (L). Data represented as mean ± s.e.m. using One-tailed unpaired t-test (A-L); *p < 0.05, **p < 0.01, ***p < 0.001; NS = not significant.

Supp Figure 8. Lipidomic profiling of Control and Gq KO islets following vehicle and NAGly treatments. PLSDA of (A) Vehicle v/s NAGly for 1 hour in WT mice (B) WT v/s NAGly for 1 hour. PL-SDA of (C) 0 hour vs 1 hour NAGly; (D) 0 hour vs UBO+NAGly; (E) 0 hour vs CpdC+NAGly in PBMC.

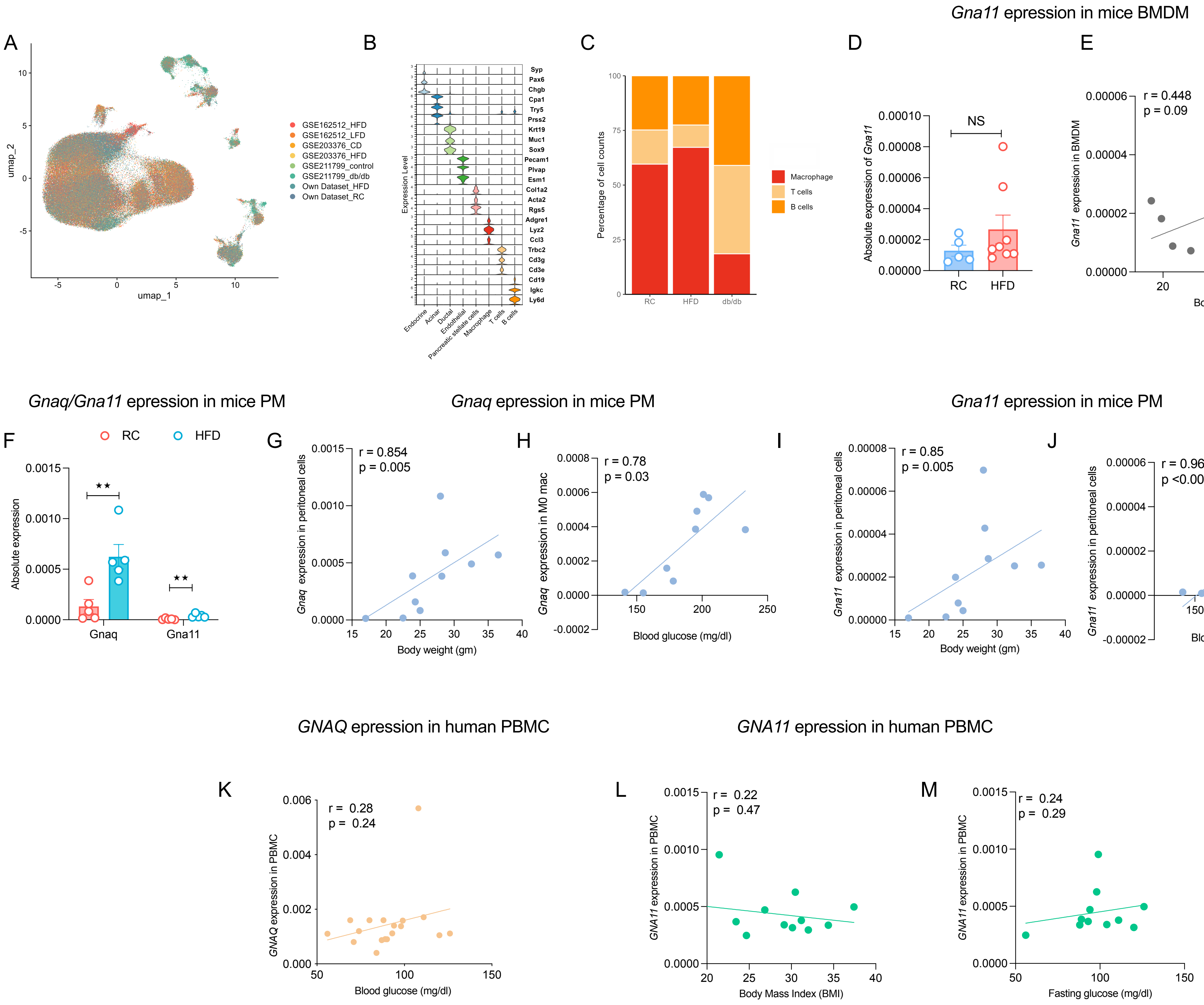

A
umap_1
umap_2
GSE162512_HFD
GSE162512_LFD
GSE203376_CD
GSE203376_HFD
GSE211799_control
GSE211799_db/db
Own Dataset_HFD
Own Dataset_RC
B
Expression Level
Syp
Pax6
Chgb
Cpa1
Try5
Prss2
Krt19
Muc1
Sox9
Pecam1
Plvap
Esm1
Col1a2
Acta2
Rgs5
Adgre1
Lyz2
Ccl3
Trbc2
Cd3g
Cd3e
Cd19
Igkc
Ly6d
Endocrine
Acinar
Ductal
Endothelial
Pancreatic stellate cells
Macrophage
T cells
B cells
C
Percentage of cell counts
RC
HFD
db/db
Macrophage
T cells
B cells
Gna11 epression in mice BMDM
D
Absolute expression of Gna11
NS
RC
HFD
E
Gna11 expression in BMDM
r = 0.448
p = 0.09
Body weight (gm)
Gnaq/Gna11 epression in mice PM
F
RC
HFD
Absolute expression
★★
Gnaq
Gna11
Gnaq epression in mice PM
G
Gnaq expression in peritoneal cells
r = 0.854
p = 0.005
Body weight (gm)
H
Gnaq expression in M0 mac
r = 0.78
p = 0.03
Blood glucose (mg/dl)
Gna11 epression in mice PM
I
Gna11 expression in peritoneal cells
r = 0.85
p = 0.005
Body weight (gm)
J
Gna11 expression in peritoneal cells
r = 0.96
p <0.0001
Blood glucose (mg/dl)
GNAQ epression in human PBMC
GNA11 epression in human PBMC
K
GNAQ expression in PBMC
r = 0.28
p = 0.24
Blood glucose (mg/dl)
L
GNA11 expression in PBMC
r = 0.22
p = 0.47
Body Mass Index (BMI)
M
GNA11 expression in PBMC
r = 0.24
p = 0.29
Fasting glucose (mg/dl)

Figure S1

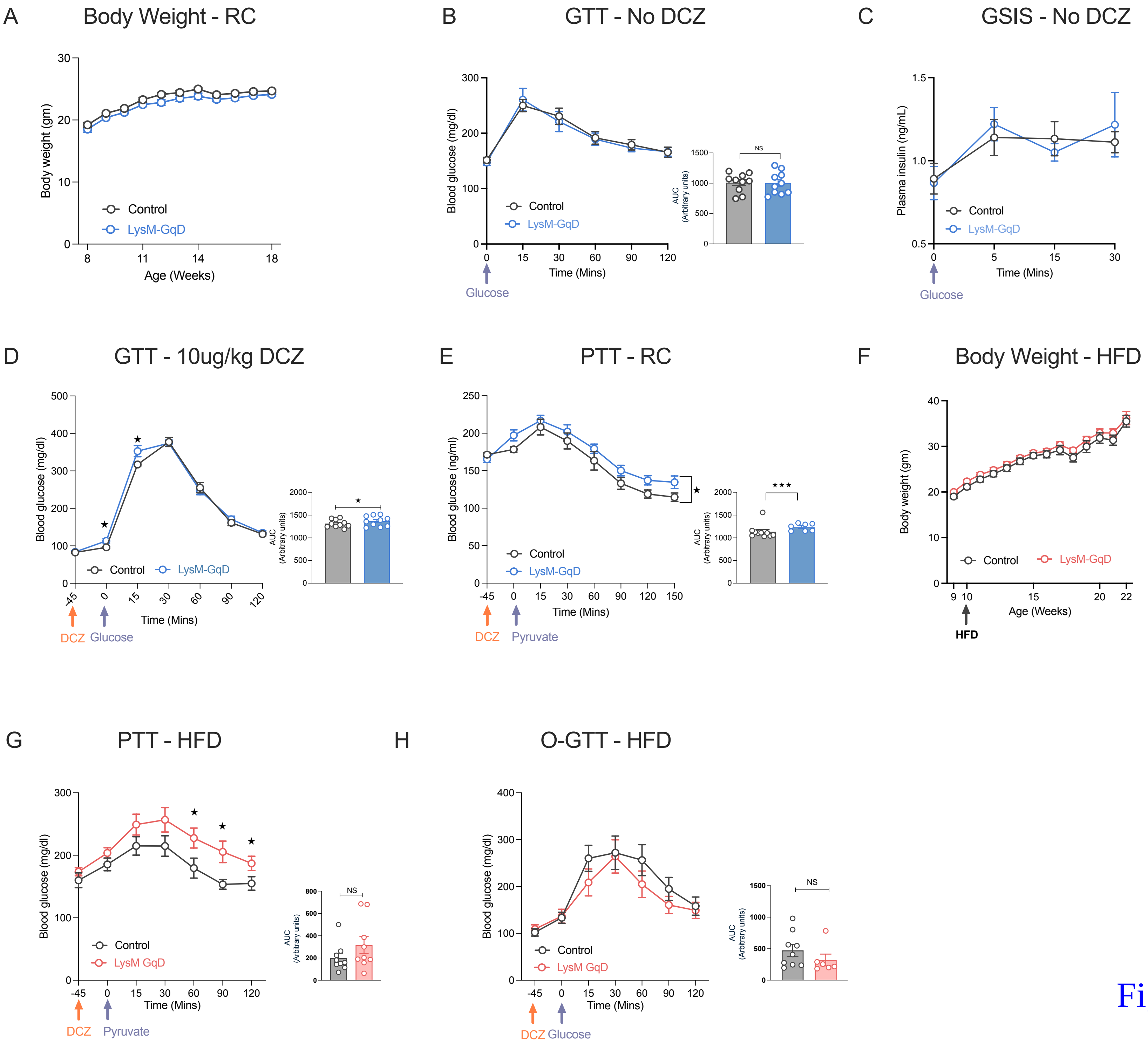
A
Body Weight - RC
Body weight (gm)
Age (Weeks)
Control
LysM-GqD
B
GTT - No DCZ
Blood glucose (mg/dl)
Time (Mins)
Glucose
AUC (Arbitrary units)
NS
C
GSIS - No DCZ
Plasma insulin (ng/mL)
Time (Mins)
Glucose
D
GTT - 10ug/kg DCZ
Blood glucose (mg/dl)
Time (Mins)
DCZ
Glucose
E
PTT - RC
Blood glucose (ng/ml)
Time (Mins)
Pyruvate
F
Body Weight - HFD
Body weight (gm)
Age (Weeks)
HFD
G
PTT - HFD
Blood glucose (mg/dl)
LysM GqD
H
O-GTT - HFD

Figure S2

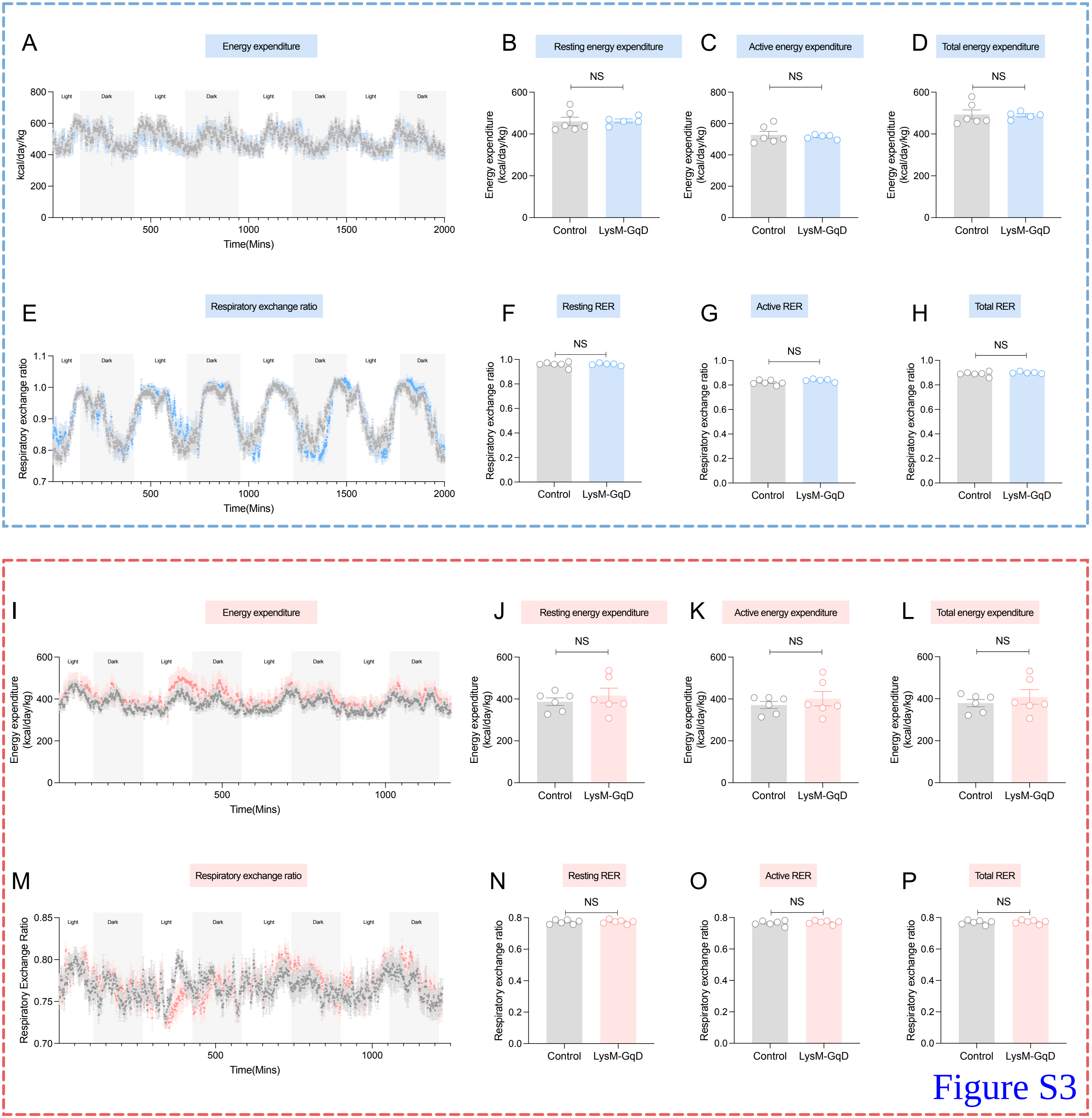
A
Energy expenditure
B
Resting energy expenditure
C
Active energy expenditure
D
Total energy expenditure
kcal/day/kg
Energy expenditure (kcal/day/kg)
Time(Mins)
Light
Dark
NS
Control
LysM-GqD
E
Respiratory exchange ratio
F
Resting RER
G
Active RER
H
Total RER
Respiratory exchange ratio
I
Energy expenditure
J
Resting energy expenditure
K
Active energy expenditure
L
Total energy expenditure
M
Respiratory exchange ratio
N
Resting RER
O
Active RER
P
Total RER
Respiratory Exchange Ratio

Figure S3

Figure S4

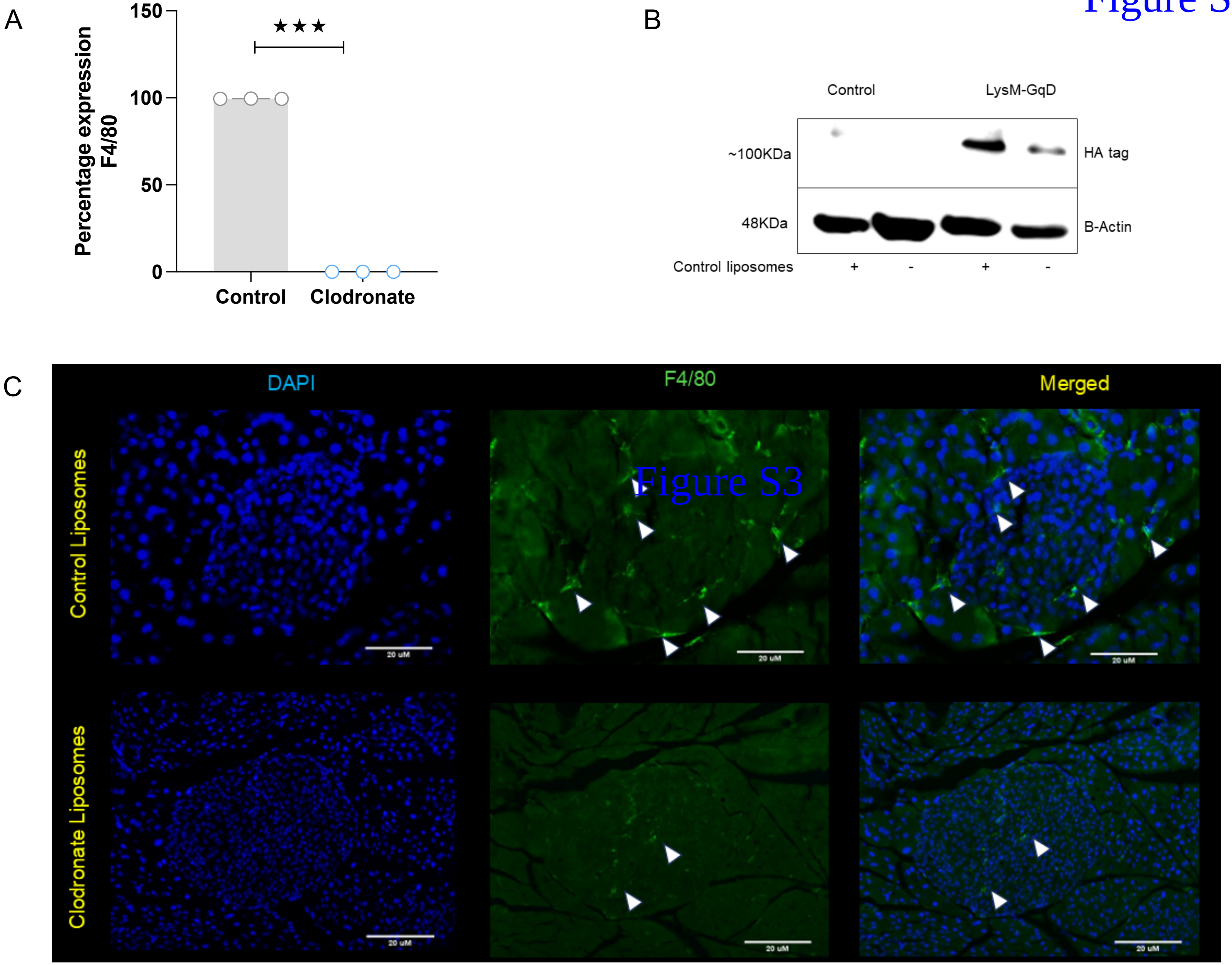

A
Percentage expression F4/80
150
100
50
0
★★★
Control
Clodronate
B
Control
LysM-GqD
~100KDa
48KDa
HA tag
B-Actin
Control liposomes
+
-
+
-
C
DAPI
F4/80
Merged
Control Liposomes
Clodronate Liposomes
20 uM
Figure S3

bioRxiv preprint doi: https://doi.org/10.1101/2025.10.29.680858; this version posted October 30, 2025. The copyright holder has placed this preprint (which was not certified by peer review) in the Public Domain. It is no longer restricted by copyright. Anyone can legally share, reuse, remix, or adapt this material for any purpose without crediting the original authors.

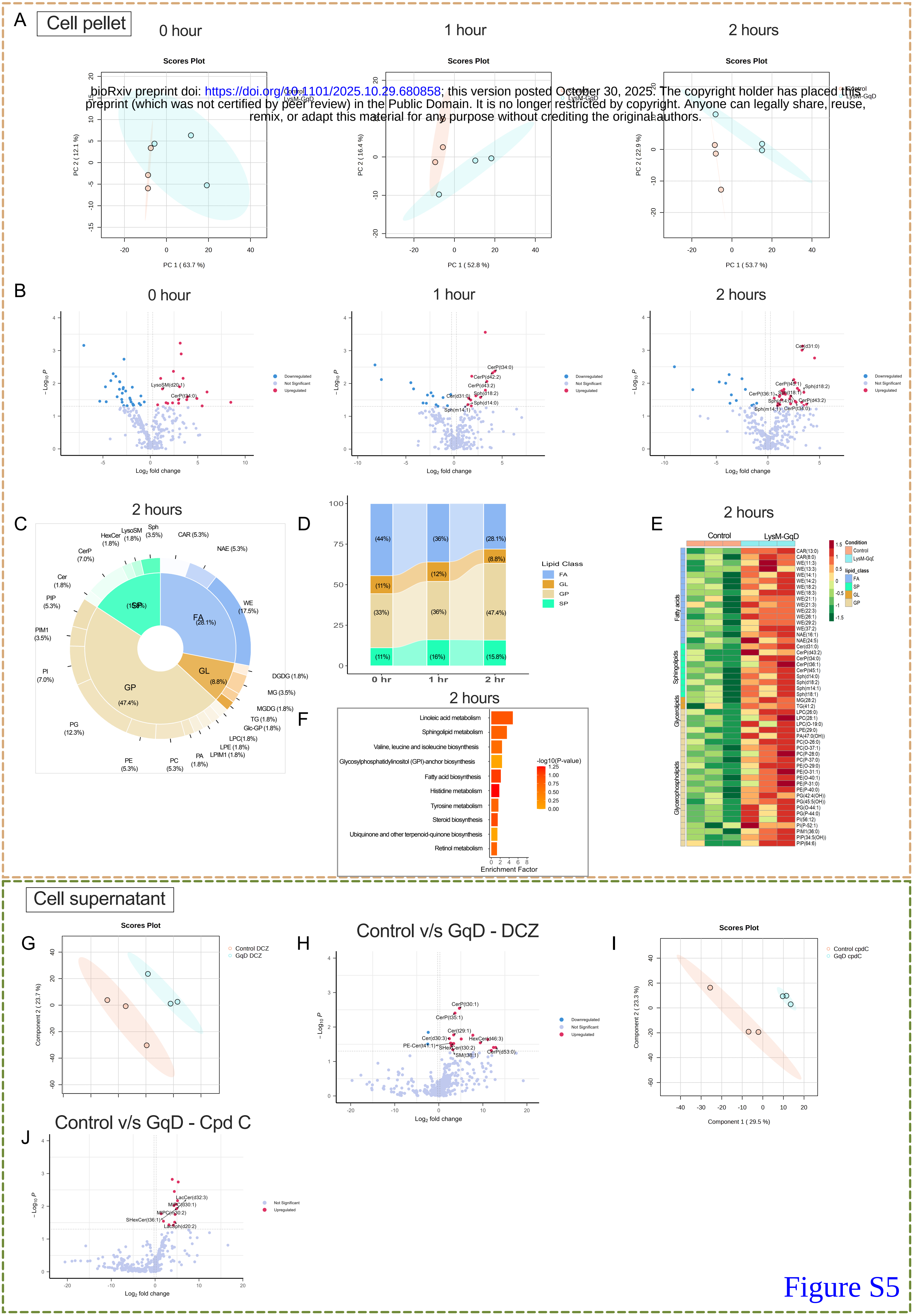

Figure S5

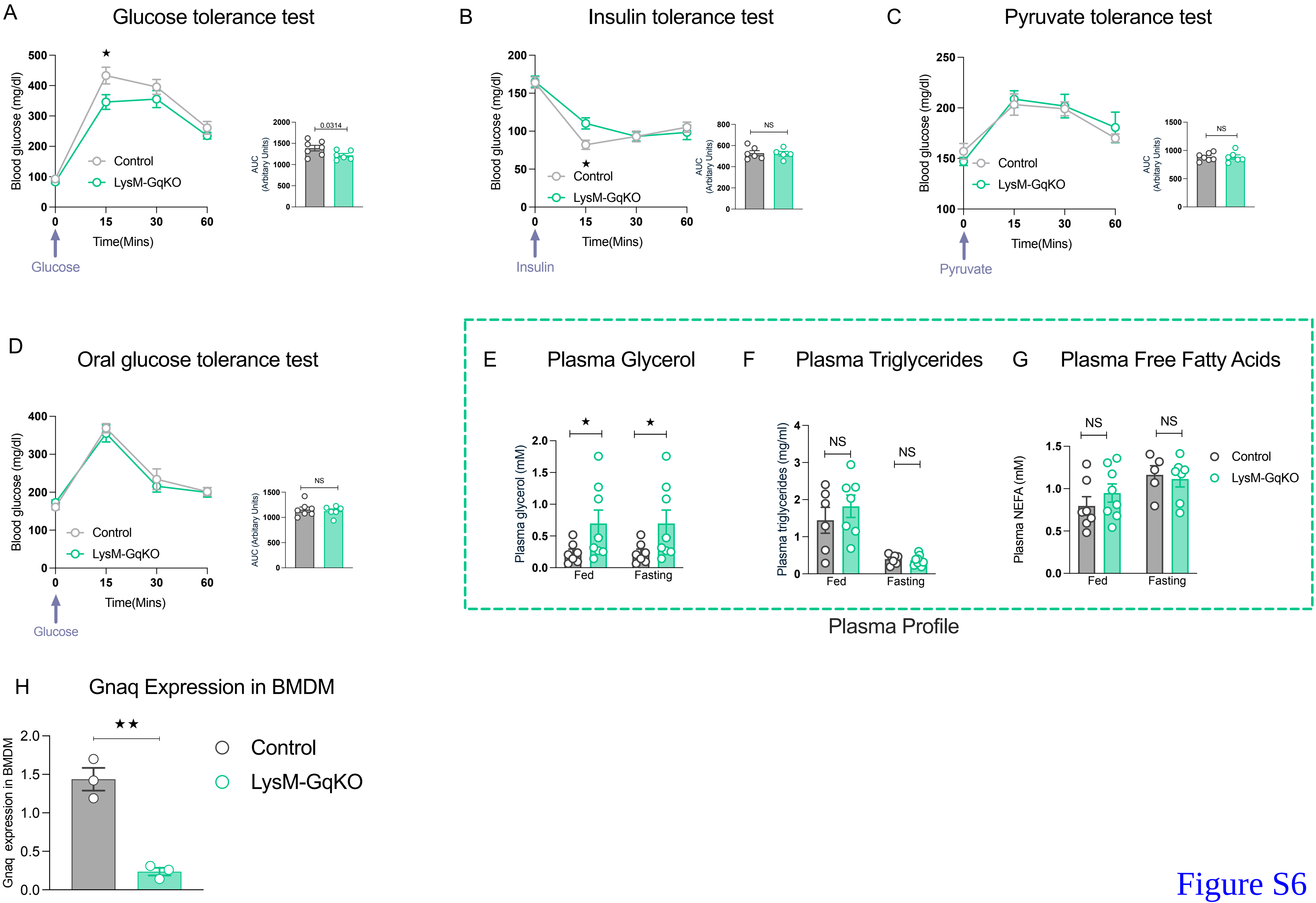
A
Glucose tolerance test
Blood glucose (mg/dl)
500
400
300
200
100
0
0
15
30
60
Time(Mins)
Glucose
Control
LysM-GqKO
AUC (Arbitary Units)
0.0314
B
Insulin tolerance test
Blood glucose (mg/dl)
Insulin
Control
LysM-GqKO
AUC (Arbitary Units)
NS
C
Pyruvate tolerance test
Blood glucose (mg/dl)
Pyruvate
Control
LysM-GqKO
AUC (Arbitary Units)
NS
D
Oral glucose tolerance test
Blood glucose (mg/dl)
Time(Mins)
Glucose
Control
LysM-GqKO
AUC (Arbitary Units)
NS
E
Plasma Glycerol
Plasma glycerol (mM)
Fed
Fasting
F
Plasma Triglycerides
Plasma triglycerides (mg/ml)
NS
NS
Fed
Fasting
G
Plasma Free Fatty Acids
Plasma NEFA (mM)
NS
NS
Fed
Fasting
Control
LysM-GqKO
Plasma Profile
H
Gnaq Expression in BMDM
Gnaq expression in BMDM
Control
LysM-GqKO

Figure S6

bioRxiv preprint doi: https://doi.org/10.1101/2025.10.29.680858; this version posted October 30, 2025. The copyright holder has placed this preprint (which was not certified by peer review) in the Public Domain. It is no longer restricted by copyright. Anyone can legally share, reuse, remix, or adapt this material for any purpose without crediting the original authors.

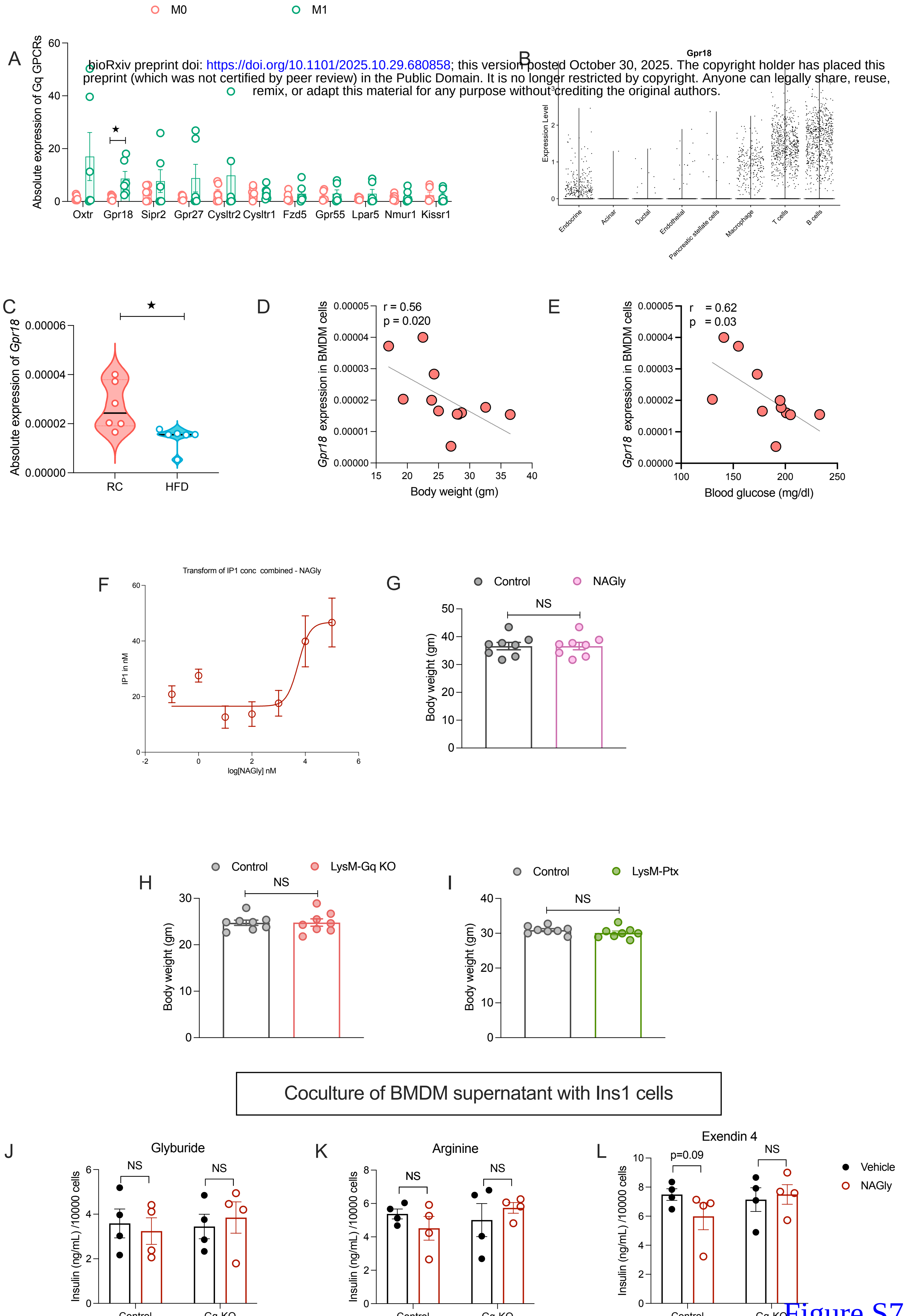

Figure S7

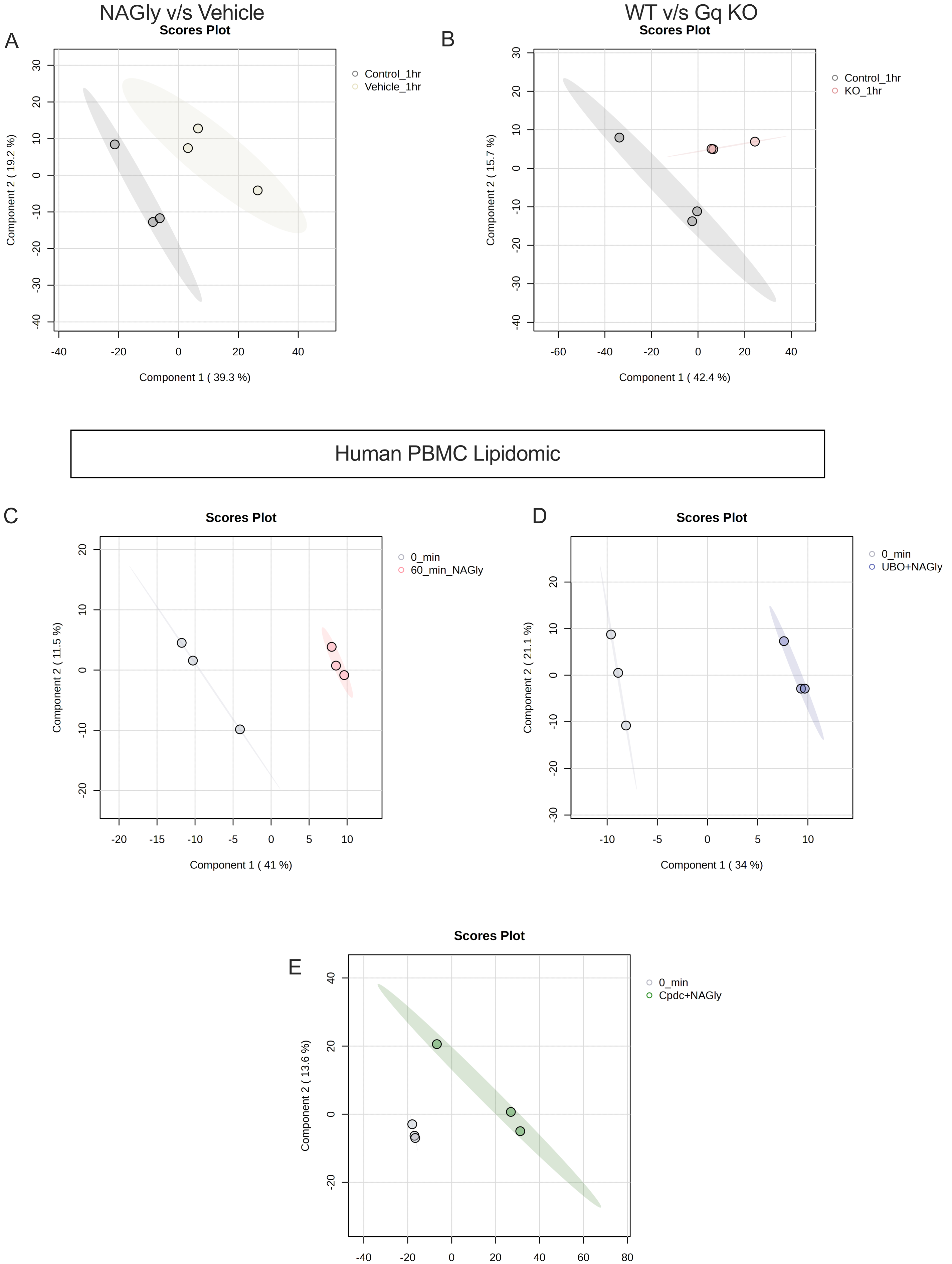
A
NAGly v/s Vehicle
Scores Plot
Control_1hr
Vehicle_1hr
Component 1 (39.3 %)
Component 2 (19.2 %)
B
WT v/s Gq KO
Scores Plot
Control_1hr
KO_1hr
Component 1 (42.4 %)
Component 2 (15.7 %)
Human PBMC Lipidomic
C
Scores Plot
0_min
60_min_NAGly
Component 1 (41 %)
Component 2 (11.5 %)
D
Scores Plot
0_min
UBO+NAGly
Component 1 (34 %)
Component 2 (21.1 %)
E
Scores Plot
0_min
Cpdc+NAGly
Component 1 (71.8 %)
Component 2 (13.6 %)

Figure S8
